# Defining crisis in families of individuals with autism spectrum disorders

**DOI:** 10.1177/1362361313508024

**Published:** 2014-11

**Authors:** Jonathan A Weiss, Aranda Wingsiong, Yona Lunsky

**Affiliations:** 1 York University, Canada; 2 Centre for Addiction and Mental Health (CAMH), Canada

**Keywords:** autism spectrum disorder, crisis, family, qualitative study

## Abstract

Parents of children diagnosed with autism spectrum disorder often report higher levels of depression, anxiety, and mental health–related issues. The combination of stressors and family adjustment difficulties can cause distress which may develop into a crisis. Understanding crisis in the family is important to mental health practice since it can serve as a guide in delivering service to at-risk families. This study investigated the subjective experience of crisis in 155 mothers of children diagnosed with autism spectrum disorder. Thematic analysis revealed that crisis is characterized by factors influencing four major areas: demands, internal capabilities, external resources, and subjective appraisal. Understanding what crisis means to families of individuals with autism spectrum disorder can help inform effective preventative and crisis services.

Parents of individuals with autism spectrum disorders (ASD) often report high levels of stress and mental health problems, associated with the challenges of caring for individuals with complex needs and with navigating multiple service sectors across the life course. Parents usually have the major obligation of caring for their child (which includes management of problematic behaviors) and balancing associated stress while overseeing the well-being of the rest of their family (Seltzer et al., 2004). Mothers of children with ASD report greater emotional problems compared to mothers of children with intellectual disability without ASD and mothers of typically developing children (Totsika et al., 2011). Similarly, mothers of adolescents and adults with ASD experience higher levels of stress and emotional problems compared to other parents (Abbeduto et al., 2004), and more frequently endorse experiences of fatigue, arguments, and stressful events (Smith et al., 2010). For many families, a buildup of stressors can result in crisis, defined in the general population as

an acute disruption of psychological homeostasis in which one’s usual coping mechanisms fail and there exists evidence of distress and functional impairment. The subjective reaction to a stressful life experience that compromises the individual’s stability and ability to cope or function. (Roberts, 2000: 516)

We know that high levels of distress are related to lower parent and child psychological well-being and can indirectly affect parenting behavior and child outcomes (Hastings and Beck, 2004; Koegel et al., 1992; Totsika et al., 2013), making studying crisis an important area of focus. We do not yet know though whether such a definition is comprehensive or representative enough to reflect the crisis experience of parents who care for individuals with ASD.

Little research exists specifically on the context of crisis within the family of individuals with ASD despite considerable research on the associated experience of stress and well-being. While many parents report positive gains in raising children with ASD (e.g. Hastings et al., 2005), stakeholder advisory groups often highlight the impact of crisis (National Autistic Society, 2006; Standing Senate Committee on Social Affairs, Science and Technology, 2007) and that services exist to support families of people with ASD in crisis (e.g. Barry and Singer, 2001; Murphy and Verdeen, 2013). Recently, White et al. (2012) explored the antecedents and consequences of crisis in families of people with ASD in a qualitative study by asking them to describe their experiences of crisis. The three most commonly endorsed precursors to crisis were the severity of a problem, a need for increased service, and problems with service providers. During or following crisis, families recalled requiring social support and more strategies to help them cope, using emergency services, and being concerned about the future of their child. While White et al. (2012) described some of the experiences of crisis in families of individuals with ASD, their analysis did not converge on an operationalized definition of crisis within the ASD context.

Multiple frameworks of family processes emphasize that coping with stressors and successful maternal adjustment is indeed possible, but that when it fails, families can experience periods of crisis. Both the Double ABCX (McCubbin and Patterson, 1983) and the Family Adjustment and Adaptation Response (FAAR) models (Patterson, 1988) suggest that the buildup of stressors in a parent’s life can be mitigated by their internal and external resources and by the meaning they apply to their situation, with successful adaptation and well-being being a possible outcome. Unlike chronic stress, crisis emphasizes an acute subjective negative experience, which threatens an individual’s functioning, values, and goals (Baum, 1990). Conversely, a crisis can occur when there is a large imbalance “emerging in the family system when the nature and/or number of demands exceed the existing capabilities of the family” (Patterson, 1988: 209).

It is important to develop a contextualized definition of crisis that reflects the lived experience of families of individuals with ASD. Such a definition can assist in identifying common variables and mechanisms that can be targets of preventative and crisis-driven intervention (Strickland, 2005). Common definitions also serve as the basis for a common understanding between clients, families, and service providers, necessary factors in a collaborative working relationship around client difficulties (Dibben and Lean, 2003). Finally, comparing an ASD-specific definition of crisis to a commonly agreed upon generic definition of crisis informs us as to the particular needs of families of people with ASD compared to the general population when crisis occurs. By defining crisis based on deconstructing and coding what families tell us, we can derive an empirically supported model of crisis, containing elements from more generic definitions but including unique aspects for those families.

The purpose of this study was to explore what crisis means to mothers of children with ASD through a thematic analysis of their answers to an open-ended question. Conducting a qualitative study can provide access to crisis-related data that are not readily observable through questionnaire report of stress or mental health problems, speaking more to the process of crisis. We examine how parent definitions of crisis compare to the common generic definition put forth by Roberts (2000) or Patterson (1988), building on them to come to a comprehensive map of the crisis experience.

## Methods

### Participants

The study involved 155 mothers, aged 26–71 years (*M* = 44.96, *SD* = 7.93), whose children were 5–48 years old (82% male, *M* = 15.31, *SD* = 7.43), out of a larger survey sample of 186 mothers who participated in the quantitative aspect of the survey. The diagnoses were as follows: Asperger syndrome (28%), autism (50%), and pervasive developmental disorder–not otherwise specified (PDD-NOS; 20%). In all, 50% of parents noted that their children had below average intellectual functioning. The vast majority of individuals with ASD were living at home (94.8%). Socioeconomic status was estimated based on the average income associated with the participants’ forward sortation area of postal codes (first three digits) using Statistics Canada’s 2006 Canadian Census (Statistics Canada, 2008). Participants’ average income ranged from CAD$25,309 to CAD$111,006, with an overall average of approximately CAD$40,762 (median = CAD$37,687; *SD* = CAD$12,352). Most participants lived in Ontario (78%), followed by Saskatchewan (8%) and British Columbia (5%). English was the first language for 91% of the sample. With respect to ethnicity, 89% of participants identified of European Canadian background. Those who responded to the open-ended question did not differ in parent age, income range, child diagnostic status, or child age, compared to those who did respond (all *p*s > .60). Approximately 5% of the sample noted that they were currently in crisis.

### Procedure

The participants were recruited through online postings and email circulation by Canadian Asperger and Autism advocacy organizations. Parents also had the opportunity to share the survey link with other parents of children with ASD; however, participation was limited to a maximum of one caregiver per family.

In this study, thematic analysis was performed on data collected from a large, online survey of parents of individuals with developmental disability in Canada (Weiss and Lunsky, 2011). In order to produce detailed data of a representative experience (Giacomini and Cook, 2000), this analysis specifically focused on the open-ended question included in the survey, which asked parents to describe what they perceive a crisis to be: “People have different ideas of what a crisis is. In your own words, what would a crisis look like for you?” This provided participants with the opportunity to respond in various ways, such as describing a previous event they identify as a crisis and/or listing characteristics they associate with crisis, making it possible to see sufficient breadth of content across observations and depth within each observation (Giacomini and Cook, 2000).

### Analysis

The parent responses from the survey varied in length, ranging from 3 to 140 words (*M* = 24.5, *SD* = 22.3). These responses were analyzed using the thematic analysis approach outlined by Braun and Clarke (2006). This approach was chosen for its applicability to a diverse set of epistemological and theoretical approaches. Its exploratory nature allows for the investigation of a broad research question and emphasizes assimilating and accommodating new themes as they emerge and develop. This was particularly useful when comparing themes to generic definitions of crisis.

Analysis involved identification and understanding of the participant perspective on crisis and extraction of themes present in their responses. Two analytical approaches were adopted to better capture and represent the information gathered from the responses (Giacomini and Cook, 2000). The frequency of relevant words used in the responses was analyzed, and a close interpretation of the text was conducted. A close reading of the responses in its entirety was done in order to obtain a better understanding and become familiar with the collected data. Each individual response was then read for present themes and coded using a qualitative software analysis tool (NVivo, Version 9). The responses were divided into meaning units (MUs) and organized into categories. The themes were reviewed, refined, and validated with consideration of their relation to the original data set. Two authors deliberated and discussed the themes, along with various approaches to data interpretation. When appropriate, individual responses were coded into multiple MUs and classified under more than one theme. Authors discussed the codes that were formulated and assigned to the themes, with the purpose of ensuring that the theme assigned to the clusters of MUs was representative not only of the cluster as a whole but also each individual MU that falls under that theme. Further computer analysis involved clustering of the codes and forming general categories and subcategories to adapt to themes as they emerged during the analysis process. While it was not possible to conduct member checking with the respondents specifically about their comments and the derived themes, as the survey was anonymous, the authors did review the MUs that were extracted with an advisory group of mothers of individuals with ASD to ensure that the generated themes were appropriately linked to anonymous content.

## Results

**Figure 1.**
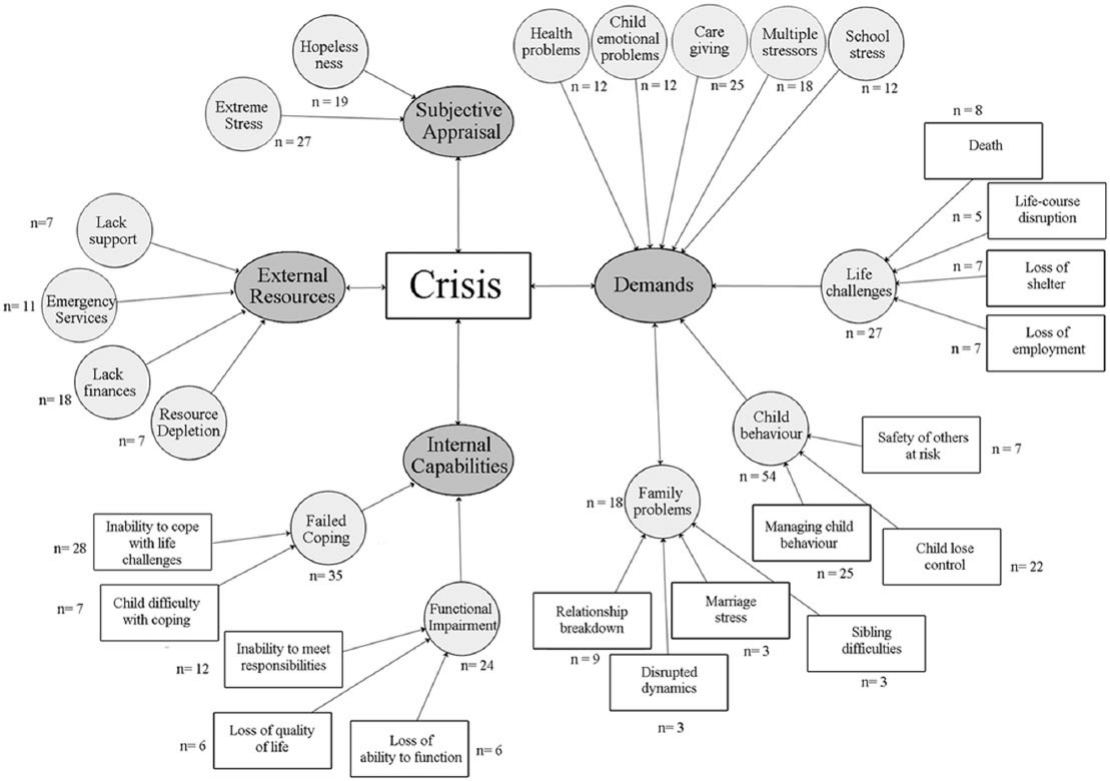
Thematic model of crisis based on survey responses from mothers of children with ASD. The figure outlines the four major themes (demands, internal capabilities, external resources, and subjective appraisal), subthemes under each main category (i.e. hopelessness and extreme stress), and the frequency of responding. ASD: autism spectrum disorder.

During the clustering stage of analysis, a decision was made to consult the definition of crisis by Roberts (2000) and the FAAR model (Patterson, 1988) in order for theory triangulation with existing social science theories (Giacomini and Cook, 2000). The emerging themes were not fully represented by either of these models independently; however, some of the elements in this analysis were similar to those presented by the two definitions. The results indicate that crisis is characterized by factors influencing four major areas: demands, internal capabilities, external resources, and subjective appraisal, similar to the core features of Roberts’ and the FAAR model’s definition of crisis. Figure 1 illustrates the breakdown of the themes and subthemes, along with the frequency of the participants identifying each one. The interactions between these factors are represented by the bidirectional arrows present in the thematic model of crisis, with the experiences of these mothers suggesting that crisis is multifaceted. We also present the age of the parent and child next to each quote, to further contextualize the findings, given our age range. As shown in Table 1, across age groups (under 13, 13–18, and 19+ years), the most commonly occurring characteristic of crisis referred to *demands*. For those aged 13 years or above, the second most common characteristic was of internal capabilities, while for the youngest group, it was issues with external resources.

**Table 1.** Major themes by child age groupings.

Themes	<13 years (n = 87)	13–18 years (n= 78)	19–25 years (n = 55)	26+ years (n = 32)
Demands (%)	52	47	44	41
External resources (%)	20	10	18	11
Subjective appraisal (%)	16	18	18	22
Internal capabilities (%)	13	24	20	26

### Demands

The responses showed that crisis arises from being faced with chronic and acute demands stemming from various factors: health problems, child emotional problems, caregiving, multiple stressors, school stress, death, life changes, child behavior, and family problems. A total of 19 examples involved a timing aspect to the definition of crisis, mostly using words that describe a chronic experience (e.g. frequent, daily lives, regularly, ongoing, and persistent). For some mothers, these demands constituted severe parent or child health–related problems (n = 12). They described it as involving “parents both ill and no one to take care of the child” (mother aged 56 years, child aged 22 years), “child [being] seriously ill” (mother aged 38, child aged 12), “mystery illness that cannot be figured out” (mother aged 40, child aged 17), and situations that lead to “imminent physical or emotional collapse” (mother aged 44, child aged 14). Central to the responses was their responsibility for their child’s health, which can include new, immediate changes to the management of medical history: “[When] my son with ASD has new medical complications (e.g. new onset seizures, or may need surgical intervention for GI [Gastrointestinal] issues)” (mother aged 42, child aged 18).

It can also include long-term, ongoing bouts with health complications:

For our son, his intermittent hard times are intense. He has bad headaches and cannot sleep … He also becomes more hypersensitive even than usual to most sensory stimuli, especially touch, sound, and proprioceptive and vestibular senses. He feels himself in turmoil, unable to sort out the mass of thoughts milling in his head, and uses images of “dark tunnel” and “tornado” to describe how he feels. Such hard times may last 10 weeks. For the past 12 years, such times have eventually ended in a grand mal seizure after which he sleeps 20 hours and almost always awakes in perfect health again. (Mother aged 39, child aged 13)

The behavioral, cognitive, and communicative deficits observed in individuals with ASD are often accompanied by co-occurring conditions (Simonoff et al., 2008). Invested in their child’s well-being, parents were also greatly occupied with their child’s behavior (n = 54) and emotional problems (n = 12). They described the difficulties they face in managing their child’s behavior (n = 25), their fears about their child losing control (n = 22), and the potential risks to the safety of others (n = 7). For some families, crisis is a combination of all three factors:

[My son] tried to bite me again. He ended up on the floor, banging his head [and] his father was holding him down. We called the ambulance [and] the police … we spent the night in the emergency [room]. We are worried that it might get to the point where we are afraid to allow him home. Then what would he do? (Mother aged 39, child aged 18)

Other mothers reported their experience of crisis as witnessing their child being impacted by serious emotional problems. Some of these responses include the following: “depression of child resulting in self-injurious behavior” (mother aged 44, child aged 22), “son having a complete mental breakdown and not knowing how to react” (mother aged 42, child aged 22), “my child not being able to deal with life … depression and anxiety running his life” (mother aged 32, child aged 14), and “suicidal tendencies” (mother aged 39, child aged 9). In certain cases, there was an overlap between behavior and emotional problems: “Daughter unable to cope with a situation and cannot understand how to deal with the situation they are in and they get upset and it keeps escalating to a point where she wants to injure herself” (mother aged 52, child aged 19).

In emphasizing these health, behavior, and emotional demands, parents also described the role such factors play in shaping caregiving demands: “All energies are devoted to attempting to soothe the child with ASD or prevent injury to sib[lings], and to prevent destruction of property. Attempts to redirect require full time supervision from early morning until late evening” (mother aged 34, child aged 17).

Crisis can also result from difficulties in the home, or family problems (n = 18), which mothers described as marriage stress (n = 3), sibling difficulties (n = 3), disrupted dynamics (n = 3), and relationship breakdown (n = 9). Some keywords included “kids fighting, family fighting” (mother aged 51, child aged 20); “breakdown in family relationships” (mother aged 39, child aged 17); and “mental breakdown and marital breakdown” (mother aged 46, child aged 14). One mother depicted the situation as the “point at which we cannot function well together as a unit” (mother aged 50, child age not reported), and another related this same issue with previously mentioned themes of child behavior and caregiving demands: “[Family] members start to get upset with one another and it becomes challenging to accomplish things such as being on time for work or even going to work because of problems with our son” (mother aged 31, child aged 11).

As these children grow older and begin to attend school, behavioral problems present in the home manifest in the school setting. Mothers described their crisis as being related to school stress (n = 12) and credit it to events such as “[receiving] frequent phone calls from school/summer daycare … that he has lost control again/ran/threatened [someone]” (mother age not reported, child aged 14), “having trouble at school or being in trouble” (mother aged 43, child aged 12), “[being] asked to remove him from a child care or school situation because they could no longer manage” (mother aged 36, child aged 12), and “bullying” (mother aged 39, child aged 10). These behavioral and emotional problems within the school setting also place additional stress on the parents to engage in the arduous feat of trying to find an appropriate and supportive learning environment for their child:

… behaviour such as hitting himself led us to remove him from public school and move him to a private school that offers CAPD [Central Auditory Processing Deficit] therapies and smaller class placement. He had been exhibiting problems with anxiety due to school pressures and was hitting himself so we went for therapy and then moved him to the new school. (Mother aged 55, child aged 28)

Mothers described their crisis as an event that led to significant changes in the family’s current way of life (n = 27). The responses falling under this theme were related to death (n = 8), life-course disruption (n = 5), loss of shelter (n = 7), and loss of employment (n = 7). Death is difficult for any family to deal with, and even more so for families of children with ASD, as it poses additional challenges to the caregivers: “Our autistic identical twin died coming up on 2 years now, and it’s hard for his autistic twin now as he’s dealing with his death now and has brought him back. That’s crisis to me” (mother aged 48, child aged 19).

Some depict crisis as major changes in their life like “change in the household (divorce, separation, death)” (mother aged 48, child aged 18), “having to move from your home to feel safe” (mother aged 42, child aged 20), “things not going the way they … have been planned” (mother aged 46, child aged 21), and “transitioning to a new school” (mother aged 51, child aged 24). It is the “lack of familiar continuity in one’s life” (mother aged 42, child aged 20). The loss of financial resources was particularly prominent:

As a disabled senior living in poverty, the insecurity of my housing is a great stress that becomes a crisis once a year. Often [Disability Supports] is the creator of crises. Having to help create programs for my son gets more difficult but there is a lack of suitable, appropriate, acceptable programs … and money is always an issue. (Mother aged 48, child aged 23)

Some mothers also described their crisis specifically as the cumulative impact of multiple stressors (n = 18). They call attention to an overload or simultaneous emergence of problems and described it, “when multiple areas of a person’s life are at a stressful point” (mother aged 41, child aged 15), when it is “difficult in all areas of your life” (mother aged 35, child aged 8), and “when things are all falling apart at once” (mother aged 46, child aged 21). A few of them listed a series of different areas where they and their families experience distress. These areas included finance, housing, health, school, and emotional problems; all themes falling under at least one of the previous subthemes were discussed.

### Internal capabilities

Mothers also associated their experience of crisis with their internal capabilities, referring to either failed coping (n = 35) or functional impairment (n = 24). Failed coping entails either the mother’s perceived inability to cope with their life challenges (n = 28) or their child’s difficulty with coping (n = 7). This is described as being faced with a situation where they are unable to deal with their difficulties. They identify crisis as occurring when they are forced to seek help from others to cope with their daily life demands because they feel limited in terms of their individual capabilities. This influences not only their daily life but also their dealings with others:

Not being able to have [past] quality of life and cope. Arguing between ourselves and being “raw” due to being a caregiver and dealing with stress of raising and dealing with daughter and all that entails on a daily basis. (Mother aged 48, child aged 20)

The failure of coping was also noted in reference to their children: “When our daughter ‘shuts down’ because of a stressor (real or imagined) in her life and refuses communication of any kind with us” (mother aged 61, child aged 42). The theme of coping extends beyond challenges in the home and includes other aspects of the individual’s life. As one mother described, “all system failure … children not coping, parents not coping, school failure, jobs compromised, [and] everything spinning out of control” (mother aged 42, child aged 20).

With regards to functional impairment, mothers reported real and/or imagined losses in their ability to function (n = 6), the fear of this leading to their inability to meet responsibilities (n = 12) and resulting in a loss of their current quality of life (n = 6). Mothers identified situations wherein they are “unable to function (i.e. unable to get out of bed, cook, [and] clean) and help [being] dependent” (mother aged 52, child aged 22) as one of crisis. It is distinguished as “negatively impact[ing] the family’s quality of life” (mother aged 41, child aged 9). They placed value not only on their functioning ability as an individual but also how loss of this ability would inhibit their ability to meet their daily responsibilities toward their families. One major concern was losing the capability to act as a caregiver to their child with ASD (mother aged 48, child aged 20). In fact, some mothers go on to describe the subtheme of disruption and change in their current quality of life as a result of weakened internal capabilities: “Crisis impacts your ability to enjoy your life in the manner that it is meant to be lived” (mother aged 70, child aged 45). Most refer not only to their lives but also the lives of their children and their entire family.

### External resources

Crisis is described as a situation where the individual and/or the entire family experiences a lack, or sudden loss, of external resources. Mothers refer to lack of support (n = 7), issues with use of emergency services (n = 11), lack of finances (n = 18), and the absolute depletion of resources available to them (n = 7). At the same time, the emphasis for these mothers was also on the *loss* of previously existing resources, as opposed to the absence of such resources.

Mothers reported experiencing difficulties getting support from their extended family and describe having a weak support network altogether. Dealing with their child(ren)’s daily caregiving demands and behaviors become an even greater challenge with the added struggle of “getting people to listen and help” (mother aged 46, child aged 18). They recognized the need for support in order to get through a crisis and closely associate this with close families and friends being unresponsive to these needs: “Family members and family friends do not wish to spend time in our home to observe the distress that the family member is experiencing or to “bother us” in times of obvious distress (father, grandparents, friends)” (mother aged 34, child aged 17).

As one mother described, “we have wonderful friends who listen to our woes, but NO ONE knocks on our door to take him out” (mother age not reported, child aged 14). This need for support also extended to the child’s formal supports, and another mother reported problems with “get[ting] my son into a good program and having to deal without team players” (mother age not reported, child aged 13).

Some mothers described crisis as a situation that is so beyond their control that they are forced to use emergency services and other government services. Some key points in their responses included worries about requiring police involvement, making 911 calls, and having to visit the emergency room. One mother saw it as “any situation where authority figures (medical, police, teachers, etc.) are involved” (mother aged 55, child aged 30). Similarly, another describes it as “[Child Protective Services] involvement again, or my threatening to call police” (mother aged 63, child aged 33). There were also concerns regarding inadequate and inefficient services which contribute to the crisis experience: “[My] son suffer[ed] an assault in his group home—[the agency] did not respond appropriately to police action [and we] finally had to contract legal services to move” (mother aged 33, child aged 9).

One major subtheme under External Resources is the lack of financial support. Mothers described fears about “loss of income” (mother aged 34, child aged 12), “bankruptcy” (mother aged 42, child aged 12; mother aged 55, child aged 33; mother aged 39, child aged 19), “losing a job or respite funding” (mother aged 37, child aged 12), and “financial failure” (mother aged 71, child aged 31). As previously discussed, parents face many different demands that come with raising a family, and many of these require sufficient income or finance:

Not being able to financially support my family. Having been sent a hydro cutoff notice last week for $600, having to pay $400+ for physio per week … $1200 for dental of an 18 year old child on the ASD spectrum … no home support [or] money. (Mother aged 50, child aged 17)

Lack or loss of this resource leads to challenges in other aspects of their family’s lives such as a lack of “affordable childcare” (mother aged 36, child aged 10), financial concerns relating to “lack of suitable, appropriate, [and] acceptable programs” (mother aged 48, child aged 23) for their child with ASD, and having “no money for food” (mother aged 41, child aged 9). Moreover, high comorbidity of psychiatric and behavioral disorders in children with ASD results in higher medical expenditure compared to children without ASD (McCarthy, 2007). One mother identified with being in a crisis, and although she had concerns about her child’s well-being and her family losing their home, she rooted these back to financial issues:

I home schooled my son and found him to be gaining extremely well. I have had to file bankruptcy; and am very afraid of losing our home which would be a huge detriment to our entire family and would throw my son in total upheaval. (Mother aged 42, child aged 12)

Some mothers specifically described crisis as the complete loss of all their available resources. Mothers discuss these challenges as occurring simultaneously: “when you have exhausted all resources … and life is difficult in all areas of your life” (mother aged 35, child aged 8). Others list a depletion of resources such as time, money, emotional strength, and basic needs (i.e. shelter). These mothers depict crisis as the moment all these necessary resources are diminished and they are at a loss as to how they can meet current and future demands.

### Subjective appraisal

In defining crisis, many mothers made a reference to their subjective appraisal of their life status and their perception of current life challenges; from their responses emerged subthemes of extreme stress and hopelessness. With regards to extreme stress (n = 27), mothers described factors they believe resulted in high stress levels, the intensity of these negative emotions, as well as how it relates to other aspects of their daily lives. They described difficulties in various areas of their lives (such as employment, health, death, marital breakdown, and finances) but with an emphasis on their emotions rather than the events themselves. Crisis was characterized by feelings of frustration, emotional instability, apathy, anxiety, and short-temperedness. As one mother described, it is the “inability to act calmly, reasonably and in a pleasant manner. It is the inability to laugh at times, sleep at times, and discuss [our] problem in a relaxed environment” (mother aged 40, child aged 18). Some described this experience as being pervasive: “ongoing stress trying to provide our son with a decent life and when I can’t stop crying” (mother aged 52, child aged 16). They also list some symptoms they associate extreme stress such as “irritability, weight gain, aggression,” as well as “having difficulty maintaining patience and [a] positive outlook in everyday life” (mother aged 49, child aged 29).

Hopelessness (n = 19) is also either directly stated or implicitly described in some of these responses. Implicitly described hopelessness was coded based on negative feelings about the future, loss of motivation, and pessimistic expectations. These are similar to the three major aspects of hopelessness as defined by the Beck Hopelessness Scale (Beck et al., 1974). Figurative language such as “when everything is falling and I am unable to pick up the pieces” (mother aged 38, child aged 12) is used to allude to this feeling. Others describe a particular instance that causes them to lose hope: “my son getting to that place where he feels he would be better off dead” (mother aged 42, child aged 18). Analysis of this subcategory demonstrates that there is a recurrent theme of “running out of options” (mother aged 46, child aged 18), not having any solutions to issues, and being uncertain about how to proceed or where to turn to for support:

When your world falls apart and there is little place to turn. Specifically, when your child tries to kill [himself] and you are “interviewed” by psychiatrists so they can decide whether your child is worth saving, (i.e. what are the chances of rehabilitation “at their age” and how hard do they want to “work”). (Mother aged 54, child aged 29)

All these factors contribute to subjective appraisals that mothers associated with experiencing crisis: “This results in my son having negative thoughts about himself as a person and my having negative thoughts about myself as a mother” (mother aged 55, child aged 21). Crisis for these mothers centered on their perspective and evaluation of their current lives, as well as the future of their children and their entire family.

## Discussion

This study investigated and qualitatively analyzed what crisis means to mothers of children with ASD by inquiring into their subjective experiences and provided a thematic map summarizing these findings. We examined a large group of comments and compared it to the current generic definitions offered by Roberts (2000) and Patterson (1988). On the basis of Roberts’ definition, crisis is an acute disruption of psychological homeostasis, characterized by failure of one’s usual coping mechanisms, by an experience of distress, and by functional impairment. The FAAR model describes crisis as an imbalance in the family system arising from the number of demands exceeding the capacities of the family. The current thematic model of crisis combines and builds on these definitions suggesting that from the view of mothers of people with ASD, crisis is composed of (a) demands, (b) internal capabilities (c) external resources, and (d) subjective appraisal. We can now more accurately define crisis an experience arising from an imbalance between the cumulative impact of numerous types of demands in one’s life (e.g. child behavior, family problems, and health problems) and the availability of external resources available to meet these demands (support, emergency services, and finances). This experience is distinguished by an interruption of one’s internal capabilities to manage (failed coping skills and functional impairment) and the negative subjective appraisal of one’s situation (of extreme stress and feelings of hopelessness). We use the term interruption as an umbrella term for the halting of internal capacities such as parent and child reported inability to cope, the family’s perceived sense of incoherence and failure to establish harmony, and the parent’s feeling of having limited capacities which propels them to seek external support.

There is indeed considerable research examining the impact of cumulative demands on parents of individuals with ASD, along with other disabilities. The application of the Double ABCX model (McCubbin and Patterson, 1983) to understand coping in families of children with ASD emphasized the “pile-up” of stressors as a source of parent distress (Bristol et al., 1988), although most studies focus on a singular stressor as a predictor of parent outcome. Frequently researched demands include the severity or type of a child’s disability (Bristol, 1987; Krauss, 1993; Totsika et al., 2011) or the child’s problem behaviors or mental health issues (internalizing and externalizing problem behaviors; Hastings, 2002). Child behavior was also the most frequent subtheme across all four major themes. This is consistent with findings from previous studies, which suggest that child behavior problems are the most consistent predictor of parental stress (Davis and Carter, 2008; Hastings, 2002).

Additional demands external to the child have also been explored, although less consistently, including education, financial resources, and health (Hauser-Cram et al., 2001; Minnes and Woodford, 2005). Understandably, there is a great deal of interest by researchers and clinicians on the predominant stressors associated with poor outcomes for families, and our study highlights that parent and child health problems, caregiving tasks, problems with the child’s school, child behavior problems, family discord, and negative life events (e.g. death of a loved one and loss of shelter or employment) are represented in the demands articulated by mothers in reference to what leads to crisis.

The failure of internal capabilities to cope with the demands is another overarching theme of crisis. This component finds support in Roberts’ (2000) description of a crisis resulting from a failure of previously used coping mechanisms and the FAAR model’s reference to the need for balance between demands and family capacities. To mothers, crisis refers more to a failure to cope and resulting loss of function, than to *how* they intend to cope. Any reference to coping was highly problem focused, which is supported by the empirical associations between problem focused coping and stress in mothers of children with ASD (Smith et al., 2008) and other developmental disabilities (Essex et al., 1999). While other cognitive strategies, such as psychological acceptance, may also have merit in helping parents of individuals with ASD cope (Blackledge and Hayes, 2006; Weiss et al., 2012), when dealing with a crisis, parents are focused on dealing with the problem at hand. Mothers may only acknowledge being in crisis when they see that there has been a loss of family member’s quality of life or adaptive function. To date, most of the parent outcome literature has focused on parent stress or mental health problems as outcomes of failed coping (Hastings, 2000; Seltzer et al., 2004; Warfield, Krauss, Hauser-Cram, Upshur, and Shonkoff, 1999; Weiss et al., 2012) although some studies have assessed outcomes of quality of life (Lee et al., 2009). The theme of the requirements to meet one’s parental responsibilities has emerged in relation to caring for children with chronic illness (Quittner et al., 1998) and warrants further exploration.

Crisis was strongly related to a failure to obtain external resources or a depletion of existing ones. Roberts’ definition and the FAAR model do not focus on the loss of external resources as a precursor to crisis. Other models of parent adjustment have noted the importance of informal and formal resources external to the family as important aspects of being part of how families cope with stressors (Bristol, 1987; McCubbin and Patterson, 1983; Perry, 2004), although these have yet to be examined in relation to crisis specifically. The presence or helpfulness of supports has been correlated to well-being in parents of individuals with ASD (Hall, 2012; Twoy et al., 2007; Wodehouse and McGill, 2009). Research is needed to examine the effects of a loss of previously existing resources on family functioning. The loss of financial supports may be particularly relevant to the discussion of crisis and speaks to the importance of creating accessible funded programs or assisting families to fund the supports required to care for individuals with ASD across the life course (Parish et al., 2010).

As noted by Roberts (2000), crisis is indeed a subjective experience, with many mothers in this study describing hopelessness and stress. Hope has been shown to be negatively related to anxiety and depression, and positively related to positive affect, in parents of children with intellectual disabilities, both in terms of their beliefs that goals can be met and that they possess the ability to plan ways to meet the goals (Lloyd and Hastings, 2009). In situations of crisis, parents experience a lack of agency to meet their problem-solving goals and do not believe that they can continue to plan effectively to deal with crisis demands. An exploration of hope in families of children with ASD has emerged in qualitative studies of the experience of diagnosis (Mulligan et al., 2012) and may be an important area of future research to support families in crisis.

As evident in the analysis, these four elements (demands, internal capabilities, external resources, and subjective appraisals) interact with each other during experiences of crisis. This speaks to the importance of a systemic level of analysis when referring to outcomes for families of people with ASD. For example, the experience of hopelessness and stress can result in crisis and can be traced back to the failure of a family’s coping mechanisms. This failure to cope could be a consequence of the inadequate access to particular resources coupled with an excessive number of child and family demands. Recent studies are beginning to examine such directionality or multivariate nature of family dynamics in ASD (Hastings et al., 2005; Totsika et al., 2013), and models founded on a contextualized definition can inform this work. For example, Totsika et al. (2013) tested the reciprocal relationships between maternal well-being and child behavior problems in families of very young children with ASD, and through cross-lagged analyses found that maternal well-being predicted child behavior problems at later time points, but that child problems did not lead to later maternal well-being. Such research is critical to discerning the directionality of effects among the constructs found in the current qualitative analysis.

Best practice guidelines in the management of crisis highlight how professionals must be able to first establish rapport and demonstrate an understanding of the precipitating events related to crisis, of the client’s perception of the event, and of the types of coping mechanisms that have failed to work, in order to develop an action plan and increase client functioning (Registered Nurses Association of Ontario, 2002). This study’s results allow families of people with ASD in crisis, or who have been in crisis in the past, to define their experience for professionals and contributes to such client-centered care. Understanding family components of crisis can help us to develop a more personalized action plan, with an ability to intervene at multiple levels, depending on the unique situation. An assessment of a caregiver’s subjective appraisals of the situation, of the kinds of internal capabilities that exist or need to be developed, of the loss of external resources or the need for additional resources, and an in-depth understanding of the sources of cumulative demands can lead us to more focused or empirically driven intervention targets. Future research is needed to examine how interventions address these components and how changes in one aspect can lead to changes in other aspects.

The current results should be interpreted with caution in consideration of study limitations. First, due to the scarcity of male participants, the sample used in this study is limited to mothers of children with ASD. No previous studies were found to suggest that there is a difference between mothers and fathers in their definition of crisis, and this is a question worth investigation. Second, this was a secondary analysis of data that originated from a large online survey which was originally designed to measure a host of family variables, including crisis. This study inquired into the subjective experience of these mothers using the open-ended questionnaire section of this survey, which provides flexibility but is also responsible for the diverse range of responses among the participants. Some mothers provided elaborate and specific examples of an event they identified as a crisis, whereas others were more general and concise in their responding. Future research could look into the subjective experience of both parents through structured interviews, encouraging elaboration in parents that provide more general responses. It would also be valuable to understand the perspectives of service providers and individuals with ASD themselves regarding what crisis means.

Despite these limitations, this study provides insight into the experiences of mothers of children with ASD. It takes a unique approach in its study of families in crisis, emphasizing the subjective experiences of the individuals and providing both depth and detail that can be overlooked by quantitative analyses. The thematic map also serves as a model for researchers interested in further investigating particular aspects of crisis that may have been neglected in the past and thus contribute to creating a clearer understanding of this issue. Having an understanding of crisis in parents of children with ASD is relevant to the practice of mental health professionals (researchers and practitioners alike) as it can serve as a guide in delivering service to at-risk families. It is especially important for the efficient delivery of service to families experiencing immediate crisis. Understanding the quality of life of children with ASD, along with their family experience, can contribute to creating changes in public policies (Zekovic and Renwick, 2003), and if geared toward the improvement of health services could benefit society as a whole. Individuals with ASD and their families often require assistance, and providing optimal service to them could serve as a guide for improving service delivery to all citizens. Additionally, a more accurate definition of crisis may lead to more uniform understanding among service providers and serve as a guideline for asking the right questions when working with families in crisis.

## References

[bibr1-1362361313508024] Abbeduto L Seltzer MM Shattuck P (2004) Psychological well-being and coping in mothers of youths with autism, down syndrome, or fragile x syndrome. American Journal on Mental Retardation 109(3): 237–254. 15072518 10.1352/0895-8017(2004)109<237:PWACIM>2.0.CO;2

[bibr2-1362361313508024] Barry LM Singer GHS (2001) A family in crisis: replacing the aggressive behavior of a child with autism toward an infant sibling. Journal of Positive Behavior Interventions 3(1): 28–38.

[bibr3-1362361313508024] Beck AT Weissman A Lester D (1974) The measurement of pessimism: the hopelessness scale. Journal of Consulting and Clinical Psychology 42: 861–865. 4436473 10.1037/h0037562

[bibr4-1362361313508024] Blackledge JT Hayes SC (2006) Using acceptance and commitment training in the support of parents of children diagnosed with autism. Child & Family Behavior Therapy 28(1): 1–18.

[bibr5-1362361313508024] Braun V Clarke V (2006) Using thematic analysis in psychology. Qualitative Research in Psychology 3(2): 77–101.

[bibr6-1362361313508024] Bristol M (1987) Mothers of children with autism or communication disorders: successful adaptation and the Double ABCX model. Journal of Autism and Developmental Disorders 17(4): 469–486. 3680150 10.1007/BF01486964

[bibr7-1362361313508024] Bristol M Gallagher JJ Schopler E (1988) Mothers and fathers of young developmentally disabled and nondisabled boys: adaptation and spousal support. Developmental Psychology 24(3): 441–451.

[bibr8-1362361313508024] Davis NO Carter AS (2008) Parenting stress in mothers and fathers of toddlers with autism spectrum disorders: associations with child characteristics. Journal of Autism and Developmental Disorders 38(7): 1278–1291. 18240012 10.1007/s10803-007-0512-z

[bibr9-1362361313508024] Dibben MR Lean M (2003) Achieving compliance in chronic illness management: illustrations of trust relationships between physicians and nutrition clinic patients. Health, Risk & Society 5(3): 241–258.

[bibr10-1362361313508024] Essex EL Seltzer MM Krauss MW (1999) Differences in coping effectiveness and well-being among aging mothers and fathers of adults with mental retardation. American Journal on Mental Retardation 104: 545–563. 10587735 10.1352/0895-8017(1999)104<0545:DICEAW>2.0.CO;2

[bibr11-1362361313508024] Giacomini MK Cook DJ (2000) User’s guides to the medical literature. American Medical Association 284(3): 357–362. 10.1001/jama.284.3.357 10891968

[bibr12-1362361313508024] Hall HR (2012) Families of children with autism: behaviors of children, community support, and coping. Issues in Comprehensive Pediatric Nursing 35(2): 111–132. 22545692 10.3109/01460862.2012.678263

[bibr13-1362361313508024] Hastings RP (2002) Parental stress and behaviour problems of children with developmental disability. Journal of Intellectual & Developmental Disability 27(3): 149–160.

[bibr14-1362361313508024] Hastings RP Beck A (2004) Practitioner review: stress intervention for parents of children with intellectual disabilities. Journal of Child Psychology and Psychiatry, and Allied Disciplines 45(8): 1338–1349. 10.1111/j.1469-7610.2004.00841.x 15482495

[bibr15-1362361313508024] Hastings RP Kovshoff H Ward NJ (2005) Systems analysis of stress and positive perceptions in mothers and fathers of pre-school children with autism. Journal of Autism and Developmental Disorders 35(5): 635–644. 16177837 10.1007/s10803-005-0007-8

[bibr16-1362361313508024] Hauser-Cram P Warfield ME Shonkoff JP (2001) Children with disability: a longitudinal study of child development and parent well-being. Monographs of the Society for Research in Child Development 66(3): 1–114. 11677873

[bibr17-1362361313508024] Koegel LK Koegel RL Hurley C (1992) Improving social skills and disruptive behavior in children with autism through self-management. Journal of Applied Behavior Analysis 25(2): 341–353. 1634427 10.1901/jaba.1992.25-341 PMC1279715

[bibr18-1362361313508024] Krauss MW (1993) Child-related and parenting stress: similarities and differences between mothers and fathers of children with disabilities. American Journal on Mental Retardation 97(4): 393–404. 8427694

[bibr19-1362361313508024] Lee GK Lopata C Volker MA (2009) Health-related quality of life of parents of children with high-functioning autism spectrum disorders. Focus on Autism and Other Developmental Disabilities 24(4): 227–239.

[bibr20-1362361313508024] Lloyd TJ Hastings R (2009) Hope as a psychological resilience factor in mothers and fathers of children with intellectual disabilities. Journal of Intellectual Disability Research 53(12): 957–968. 19744261 10.1111/j.1365-2788.2009.01206.x

[bibr21-1362361313508024] McCarthy J (2007) Children with autism spectrum disorders and intellectual disability. Current Opinion in Psychiatry 20(5): 472–476. 17762590 10.1097/YCO.0b013e32821f6095

[bibr22-1362361313508024] McCubbin HI Patterson JM (1983) The family stress process: the Double ABCX model of adjustment and adaptation. In: McCubbin HI Sussman MB Patterson JM (eds) Social Stress and the Family. New York: The Hayworth Press Inc., pp. 7–38.

[bibr23-1362361313508024] Minnes P Woodford L (2005) Well-being in aging parents caring for an adult with a developmental disability. Journal on Developmental Disabilities 11: 48–66.

[bibr24-1362361313508024] Mulligan J MacCulloch R Good B (2012) Transparency, hope, and empowerment: a model for partnering with parents of a child with autism spectrum disorder at diagnosis and beyond. Social Work in Mental Health 10(4): 311–330.

[bibr25-1362361313508024] Murphy CM Verden CE (2013) Supporting families of individuals with autism spectrum disorders: developing a university-based respite care program. Journal of Positive Behavior Interventions 15(1): 16–25.

[bibr26-1362361313508024] National Autistic Society (2006) Advocacy and autism [Fact sheet]. Available at: http://www.autism.org.uk/Living-with-autism/Adults-with-autism-or-Asperger-syndrome/Advocacy-and-autism.aspx (accessed 16 February 2013).

[bibr27-1362361313508024] Parish SL Rose RA Swaine JG (2010) Income and asset poverty of parents of children and adults with developmental disabilities: a comparison of age cohorts across the lifespan. Journal of Intellectual & Developmental Disability 35(4): 235–243. 21117881 10.3109/13668250.2010.519331

[bibr28-1362361313508024] Parish SL Seltzer MM Greenberg JS Floyd FJ (2004) Economic implications of caregiving at midlife: Comparing parents of children with developmental disabilities to other parents. Mental Retardation 42: 413–426. 15516174 10.1352/0047-6765(2004)42<413:EIOCAM>2.0.CO;2

[bibr29-1362361313508024] Patterson JM (1988) Families experiencing stress: I. The Family Adjustment and Adaptation Response model: II. Applying the FAAR model to health-related issues for intervention and research. Family Systems Medicine 6(2): 202–237.

[bibr30-1362361313508024] Perry A (2004) A model of stress in families of children with developmental disabilities: clinical and research applications. Journal on Developmental Disabilities 11(1): 1–16.

[bibr31-1362361313508024] Quittner AL Espelage DL Opipari LC (1998) Role strain in couples with and without a child with chronic illness: associations with marital satisfaction, intimacy, and daily mood. Health Psychology 17(2): 112–124. 9548702 10.1037//0278-6133.17.2.112

[bibr32-1362361313508024] Registered Nurses Association of Ontario (2002). Client centred care. Toronto, Canada: Registered Nurses Association of Ontario [Online]. Available: http://www.rnao.org/bestpractices

[bibr33-1362361313508024] Roberts AR (2000) Crisis Intervention Handbook: Assessment, Treatment, and Research. 2nd ed. New York: Oxford University Press.

[bibr34-1362361313508024] Seltzer MM Shattuck P Abbeduto L (2004) Trajectory of development in adolescents and adults with autism. Mental Retardation and Developmental Disabilities Research Reviews 10(4): 234–247. 15666341 10.1002/mrdd.20038

[bibr35-1362361313508024] Simonoff E Pickles A Charman T (2008) Psychiatric disorders in children with autism spectrum disorders: prevalence, comorbidity, and associated factors in a population-derived sample. Journal of the American Academy of Child and Adolescent Psychiatry 47(8): 921–929. 18645422 10.1097/CHI.0b013e318179964f

[bibr36-1362361313508024] Smith L Hong J Seltzer MM (2010) Daily experiences among mothers of adolescents and adults with autism spectrum disorder. Journal of Autism and Developmental Disorders 40(2): 167–178. 19655239 10.1007/s10803-009-0844-y PMC2826850

[bibr37-1362361313508024] Smith LE Seltzer MM Tager-Flusberg H (2008) A comparative analysis of well-being and coping among mothers of toddlers and mothers of adolescents with ASD. Journal of Autism and Developmental Disorders 38(5): 376–889. 10.1007/s10803-007-0461-6 PMC2831472 17924181

[bibr38-1362361313508024] Standing Senate Committee on Social Affairs, Science and Technology (2007) Pay now or pay later: autism families in crisis. Final report on the enquiry on the funding for the treatment of autism. Available at: http://www.parl.gc.ca/39/1/parlbus/commbus/senate/com-e/soci-e/rep-e/repfinmar07-e.pdf (accessed 16 February 2013).

[bibr39-1362361313508024] Statistics Canada (2008) 2006 Profile for Canada, provinces, territories and forward sortation areas. Statistics Canada catalogue no. 94-581-XCB2006003. Available at: http://www5.statcan.gc.ca/bsolc/olc-cel/olc-cel?catno=94-581-X2006003&lang=eng (accessed 16 February 2013).

[bibr40-1362361313508024] Strickland OL (2005) Measurement implications when developing and implementing interventions. Journal of Nursing Measurement 13(3): 171–173. 16605040

[bibr41-1362361313508024] Totsika V Hastings RP Emerson E (2011) Behavior problems at 5 years of age and maternal mental health in autism and intellectual disability. Journal of Abnormal Child Psychology 39(8): 1137–1147. 21710136 10.1007/s10802-011-9534-2

[bibr42-1362361313508024] Totsika V Hastings RP Emerson E (2013) Is there a bidirectional relationship between maternal well-being and child behavior problems in autism spectrum disorders? Longitudinal analysis of a population-defined sample of young children. Autism Research. Epub ahead of print 21 February 2013. DOI: 10.1002/aur.1279. 23436803

[bibr43-1362361313508024] Twoy R Connolly PM Novak JM (2007) Coping strategies used by parents of children with autism. Journal of American Academy of Nurse Practitioners 19(5): 251–260. 10.1111/j.1745-7599.2007.00222.x 17489958

[bibr44-1362361313508024] Warfield ME Krauss MW Hauser-Cram P Upshur CC Shonkoff JP (1999) Adaptation during early childhood among mothers of children with disabilities. Journal of Developmental & Behavioral Pediatrics 20(1): 9–16. 10071940 10.1097/00004703-199902000-00002

[bibr45-1362361313508024] Weiss JA Cappadocia MC MacMullin J A Viecili MA Lunsky Y (2012) Psychological acceptance and empowerment as mediators of the impact of problem behaviour in children with autism spectrum disorders on parent mental health. Autism: The International Journal of Research and Practice 16: 261–274. 22297202 10.1177/1362361311422708

[bibr46-1362361313508024] Weiss JA Lunsky Y (2011) The brief family distress scale: a measure of crisis in caregivers of individuals with autism spectrum disorders. Journal of Child and Family Studies 20(4): 521–528.

[bibr47-1362361313508024] White SE McMorris C Weiss JA (2012) The experience of crisis in families of individuals with autism spectrum disorder across the lifespan. Journal of Child and Family Studies 21(3): 457–465.

[bibr48-1362361313508024] Wodehouse G McGill P (2009) Support for family carers of children and young people with developmental disabilities and challenging behaviour: what stops it being helpful? Journal of Intellectual Disability Research 53(7): 644–653. 19298502 10.1111/j.1365-2788.2009.01163.x

[bibr49-1362361313508024] Zekovic B Renwick R (2003) Quality of life for children and adolescents with developmental disabilities: review of conceptual and methodological issues relevant to public policy. Disability & Society 18(1): 19–34.

